# Decoding NOTCH1: From T-Cell Development Guardian to Driver of Pediatric T-Cell Lymphoblastic Lymphoma

**DOI:** 10.3390/ijms27042083

**Published:** 2026-02-23

**Authors:** Fran Leijnen, Tim Lammens

**Affiliations:** 1 Department of Internal Medicine and Pediatrics, Ghent University, 9000 Ghent, Belgium; fran.leijnen@ugent.be; 2 Department of Pediatric Hematology-Oncology and Stem Cell Transplantation, Ghent University Hospital, 9000 Ghent, Belgium; 3 Cancer Research Institute Ghent, 9000 Ghent, Belgium

**Keywords:** T-cell lymphoblastic lymphoma, *NOTCH1* mutations, *NOTCH1* gene fusions, targeted therapy, risk stratification, molecular genetics, pediatric hematology, pediatric oncology

## Abstract

T-cell lymphoblastic lymphoma (T-LBL) is an aggressive malignancy of immature T-cells accounting for a substantial proportion of pediatric non-Hodgkin lymphoma cases. Current chemotherapeutic regimens achieve five-year event-free survival rates of 80–90%, yet relapse occurs in approximately 20% of patients and remains a major therapeutic challenge. This underscores the need for improved, molecularly informed treatment strategies. Recent genomic profiling has highlighted the central role of *NOTCH1* signaling in T-LBL pathogenesis. *NOTCH1*, a transmembrane receptor critical for T-cell differentiation and maturation, requires tightly regulated activation during normal thymocyte development. Dysregulated signaling disrupts this balance, driving aberrant proliferation and impaired differentiation, characteristics of malignant transformation. While activating mutations have long been recognized as key oncogenic events, the recent identification of recurrent *NOTCH1* translocations, associated with adverse outcomes, reveals an additional mechanism of pathway activation. These findings reinforce *NOTCH1* as a pivotal oncogenic hub in T-cell malignancies and a compelling target for therapeutic intervention. This review synthesizes current insights into the molecular landscape of pediatric T-LBL, with a focus on the biological and clinical implications of *NOTCH1* mutations and translocations. Furthermore, we examine emerging approaches to therapeutically exploit aberrant *NOTCH1* signaling for the more precise and effective treatment of this disease and formulate outstanding research questions.

## 1. Introduction

T-cell lymphoblastic lymphoma (T-LBL) is a rare and aggressive hematologic malignancy that arises from immature T-cells. It represents the second most common subtype of non-Hodgkin lymphoma (NHL) in children and adolescents, accounting for 25–35% of all pediatric NHL cases [1,2,3]. The median age at diagnosis ranges from 7 to 10.5 years, and the condition shows a marked male predominance, with a reported male-to-female ratio of approximately 2.5:1 [1,2,4,5,6]. T-LBL typically presents as a large anterior mediastinal mass, frequently accompanied by substantial pleural or pericardial effusions [1,2]. This representation often results in severe clinical manifestations, including shortness of breath, cough, stridor, acute respiratory distress and superior vena cava syndrome [1,2,6]. In addition to mediastinal involvement, lymphoid organs such as the thymus and lymph nodes, as well as extranodal sites including the pleura, bone marrow, and central nervous system, may be affected [1,4]. The infiltrating cells are typically medium-sized lymphoblasts with scant cytoplasm and finely dispersed chromatin, which is characteristic of T-LBL morphology [1,4]. When T-LBL is suspected, biopsies should be obtained for the diagnostic workup, either from tissue lesions or malignant effusions [1,2,4]. Histopathological evaluation, supported by immunophenotyping through flow cytometry or immunohistochemistry, is essential for diagnostic confirmation [1,2,4]. The defining immunophenotypic markers for T-LBL include both markers of the T-lineage (CD3, CD2, CD4, CD5, CD7, CD8) and markers of immaturity (TdT, CD99, CD34, CD1a) [1,2,3,4,6,7]. After diagnosis, patients are staged to assess disease extent, although pediatric treatment protocols are generally uniform across stages. Staging is most commonly performed according to the St. Jude/Murphy system or the International Pediatric Non-Hodgkin Lymphoma Staging System [8,9]. Briefly, stage I involves a single lymph node region or lymphoid organ; stage II includes two or more lymph node regions on one side of the diaphragm; stage III affects lymph node regions on both sides of the diaphragm; and stage IV indicates dissemination to the central nervous system or bone marrow [8,9]. Staging is performed using bone marrow aspiration or biopsy, lumbar puncture, physical examinations and imaging studies [1,10]. Bilateral bone marrow samples are analyzed morphologically and by immunophenotyping [1,10]. Lumbar punctures are used to determine cerebrospinal fluid cell counts and cytology [1,10]. Imaging typically includes chest X-ray and ultrasound to evaluate the abdomen, lymph nodes, testes, and potential pleural and pericardial effusions [1,10]. MRI or CT is performed when mediastinal masses, bone lesions, or neurological symptoms are suspected [1,10]. PET/CT can be valuable for initial staging, especially in cases with mediastinal involvement, but should not delay treatment initiation [10].

A central and ongoing debate in the field of T-cell malignancies concerns whether T-LBL and its leukemic counterpart, T-cell acute lymphoblastic leukemia (T-ALL), represent distinct pathological entities or constitute different clinical manifestations of a single disease spectrum, as both diseases originate from the malignant transformation of thymocytes [11]. The current World Health Organization (WHO) classification and the International Lymphoma Study Group (ILSG) categorize these entities together under the term T-lymphoblastic leukemia/lymphoma (T-LBLL), reflecting their indistinguishability in morphology and immunophenotype [7,12,13,14,15]. Historically, the diagnostic distinction between T-ALL and T-LBL has been based primarily on the degree of bone marrow involvement, with infiltration exceeding 25% defining T-ALL and less than 25% defining T-LBL [1]. However, emerging genomic and transcriptome data challenges this traditional model, suggesting that T-ALL and T-LBL may constitute biologically discrete entities rather than clinical variants of a single disease process [14].

Given the similarities between T-LBL and T-ALL, T-LBL’s current therapeutic approach is largely based on a backbone of T-ALL treatment protocols, derived from the Berlin–Frankfurt–Münster (BFM) Study Group, resulting in a 5-year event-free survival rate of 80–90% [10,16,17]. The treatment schedule usually involves a 9-week induction, an 8-week consolidation and a 7-week re-induction treatment followed by oral maintenance for a total therapy duration of two years [10,16]. The treatment backbone generally consists of an eight-drug regimen (prednisone, vincristine, daunorubicin, L-asparaginase, cyclophosphamide, cytarabine, 6-mercaptopurine and methotrexate) as the induction phase, followed by consolidation with high-dose systemic methotrexate [16]. Maintenance therapy consists of daily oral 6-mercaptopurine and weekly low-dose methotrexate [16]. This approach resulted in a 5-year overall survival probability of 90% in the NHL-BFM 90 trial [16]. In the following EURO-LB 02 trial, prednisone was replaced by dexamethasone during induction, resulting in a 5-year event-free survival rate of 82% [17]. The lower survival rate is likely due to the increased toxicity associated with dexamethasone [17]. Recently, the LBL-2018 trial opened. This protocol builds upon the NHL-BFM 90 backbone, similar to the preceding EURO-LB 02 trial, and introduces two major innovations [1,10]. First, it compares a shortened 14-day dexamethasone course with the conventional 21-day prednisone regimen during induction to evaluate whether this modification can reduce the cumulative incidence of central nervous system relapses [1,10]. Second, it is the first pediatric T-LBL trial to incorporate molecular characteristics, specifically the *NOTCH1* and *FBXW7* mutational status, into risk stratification [1,10].

Despite achieving these encouraging survival rates, outcomes for patients who relapse remain dismal. Approximately 20% of patients will experience relapse, and survival rates after relapse are only 10–30% [1,2,4,18]. The standard salvage approach consists of re-induction chemotherapy, followed by a hematopoietic stem cell transplantation in patients who achieve second remission [19]. However, these strategies have limited efficacy, as relapsed disease often exhibits resistance to conventional chemotherapeutics. Moreover, a major limitation in T-LBL management is that front-line therapy still depends on intensive multi-agent chemotherapy, with no approved targeted therapies currently available. This highlights the urgent need for more precise and less toxic therapeutic strategies; however, achieving this will require deeper insight into the molecular pathobiology of T-LBL. Even though advances are being made in molecular profiling, significant gaps remain in our understanding of the genetic landscape of pediatric T-LBL. These gaps are largely due to the rarity of the disease and the frequent need to initiate treatment before sufficient diagnostic material can be collected for comprehensive genomic analyses [7,20]. Nevertheless, several recurrent alterations have emerged across several cell functions. These alterations collectively drive oncogenesis by promoting cell proliferation, disrupting cell cycle control, and altering epigenetic regulation. The following section highlights the most frequently mutated genes and dysregulated pathways.

## 2. Dysregulated Pathways in T-LBL

The largest study to date, conducted by Bontoux et al., analyzed 342 T-LBL cases, including 156 adult patients and 186 pediatric patients, using a 105-targeted gene panel [21]. The study delineated frequent alterations in *NOTCH1* (52%) and *FBXW7* (24%), alongside the recurrent disruption of cell cycle regulators, including *CDKN2A* deletions (50%) and occasional *TP53* mutations (7%) [21]. Complementary data from Khanam et al., who performed whole-exome sequencing of 16 pediatric T-LBL patients, provided additional prevalence estimates for *NOTCH1* (63%), *FBXW7* (25%), and *CDKN2A* (75%) [22]. *CDKN2B* deletions were observed in 61% of cases [22]. Furthermore, Bonn et al. analyzed a cohort of 116 pediatric patients and reported *NOTCH1* mutations in 60% of patients, which were associated with a favorable prognosis (5-year pEFS of 84% ± 5% vs. 66% ± 7%; *p* = 0.021) [23]. *FBXW7* mutations were detected in 18% of patients, and 15% harbored mutations in both genes, a combination that was also linked to favorable prognosis [23]. Beyond cell cycle control, epigenetic regulators, such as *PHF6* (22%), *EZH2* (13%) and *KMT2D* (14%), are recurrently altered, supporting a role for epigenetic deregulation in lymphomagenesis [21]. Notably, *KMT2D* mutations were associated with an unfavorable prognosis as they confer a significantly higher cumulative incidence of relapse (cumulative incidence of relapse 47% ± 17% vs. 14% ± 3%; *p* = 0.0064) [22]. Key signaling pathways, such as *JAK*-*STAT* and *PI3K*-*AKT*, are also frequently affected. Alterations in the *JAK*-*STAT* pathway occur in 33% of cases, while mutations in *PIK3CA* (7–9%) and *PTEN* (15–16%) were also observed, both promoting survival signaling [21,22]. Clinically, *PTEN* mutations have been associated with poor prognosis in pediatric T-LBL (5-year pEFS of 59% ± 12% vs. 82% ± 4%; *p* = 0.014) [24]. Similarly, the *JAK*-*STAT* pathway is hyperactivated in T-LBL patients with a poor prognosis [25]. In addition to these gene-level alterations, large-scale chromosomal aberrations (>20 Mb) are observed in approximately 39% of cases, as shown by Kroeze et al., who performed a SNP-array-based copy number profiling of 59 pediatric T-LBL patients [26]. Recurrent gains involve chromosomes 10, 17, and 20, while loss of heterozygosity on chromosome 6q (LOH 6q) occurs in about 13–16% of cases and is associated with unfavorable prognosis (pEFS 27% ± 9% vs. 86% ± 3%; *p* < 0.0001) [22,23,26]. A concise overview of prevalence estimates, prognostic relevance and references is provided in Table 1.

While this review focuses specifically on pediatric T-LBL, Bontoux et al. combined data from both pediatric and adult cases without distinguishing age-specific genetic features. Importantly, their findings are consistent with the results from pediatric-specific studies, supporting their relevance to the pediatric population.

**Table 1:** Summary of genetic alterations reported in pediatric T-cell lymphoblastic lymphoma (T-LBL), including their prevalence, prognostic associations, and corresponding references.

Biological Process	Genetic Alteration	Prevalence (%)	Prognostic Association	Ref.
*NOTCH1* pathway	*NOTCH1* ^MT^	52–63	Favorable prognosis	[21,22,23]
	*FBXW7* ^MT^	18–25	No prognostic relevance	[21,22,23]
	*NOTCH1*^MT^/*FBXW7*^MT^	15	Favorable prognosis	[23]
Cell cycle regulation	*CDKN2A* ^DEL^	50–75	No prognostic relevance/Unclear	[21,22]
	*CDKN2B* ^DEL^	61	No prognostic relevance/Unclear	[22]
	*TP53* ^MT^	7	Unclear	[21]
Epigenetic regulation	*PHF6* ^MT^	22	Favorable prognosis	[21]
	*EZH2* ^MT^	13	Not mentioned	[21]
	*KMTD2* ^MT^	14	Unfavorable prognosis	[21,22]
Survival signaling	*JAK*-*STAT* pathway^MT^	33	Unfavorable prognosis	[21,25]
	*PIK3CA* ^MT^	7–9	Not mentioned	[21,22]
	*PTEN* ^MT^	15–16	Unfavorable prognosis	[21,22,24]
Chromosomal alterations	LOH6q	13–16	Unfavorable prognosis	[22,23,26]

## 3. *NOTCH1*: From Development to Malignancy

Among all the molecular alterations, the *NOTCH1* pathway is the most affected in T-LBL, underscoring the central role of this pathway in T-cell transformation and indicating that *NOTCH1* can be considered a putative driver of T-LBL [21,22]. To understand the impact of *NOTCH1* alterations in T-LBL, it is essential to first consider the physiological role of *NOTCH1* signaling in hematopoiesis and T-cell development.

### 3.1. The NOTCH Protein Family: Structure and Functional Overview

**Figure 1:**
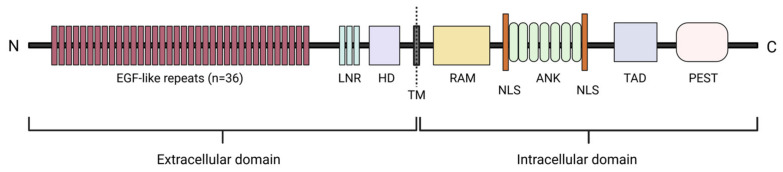
A schematic representation of the multidomain architecture of the NOTCH1 protein. EGF, epidermal growth factor; LNRs, Lin12–Notch repeats; HD, heterodimerization domain; TM, transmembrane domain; RAM, RBP-Jκ-associated module domain; ANK, Ankyrin repeats; NLS, nuclear localization signal; TAD, transactivation domain; PEST, proline–glutamate–serine–threonine-rich domain. Created with BioRender.com (https://biorender.com, accessed on 23 December 2025).

The NOTCH protein family comprises highly conserved single-pass type I transmembrane receptors that play a crucial role in regulating cell fate decisions during the development of various cell lineages, including self-renewal, differentiation, apoptosis and proliferation [27,28,29]. In mammals, four NOTCH receptors (NOTCH1-4) and five ligands (Jagged1, Jagged2, and Delta-like 1, 3, and 4) have been identified [28,30]. NOTCH ligands can interact with multiple receptors but show context-dependent preferences; for example, during T-cell development in the thymus, Delta-like 4 is the primary ligand activating NOTCH1. All NOTCH receptors share a conserved multidomain structure [31]. The extracellular region contains 29–36 tandem epidermal growth factor (EGF)-like repeats responsible for ligand binding, followed by a negative regulatory region (NRR) (Figure 1) [28,29,31]. This NRR consists of three cysteine-rich Lin12–Notch repeats (LNRs) and a heterodimerization domain (HD), which together maintain receptor stability in the absence of ligand interaction to prevent ligand-independent receptor activation [28,29,31]. The intracellular domain comprises several key modules: the RBP-Jκ-associated module (RAM) domain, seven Ankyrin repeats (ANK), two nuclear localization signals (NLSs), a transactivation domain (TAD), and a proline–glutamate–serine–threonine-rich (PEST) domain (Figure 1) [28,31]. The RAM domain and Ankyrin repeats are crucial for downstream signal transduction, as they mediate interactions with transcriptional regulators [28,32]. NLSs are important for actively transporting the NOTCH intracellular domain (NICD) into the nucleus, while the TAD is responsible for promoting target gene transcription [31]. The PEST domain contains multiple phosphorylation sites that control NICD stability through ubiquitin-mediated degradation, thereby regulating NOTCH protein turnover [28,29].

### 3.2. Canonical NOTCH Signaling

**Figure 2:**
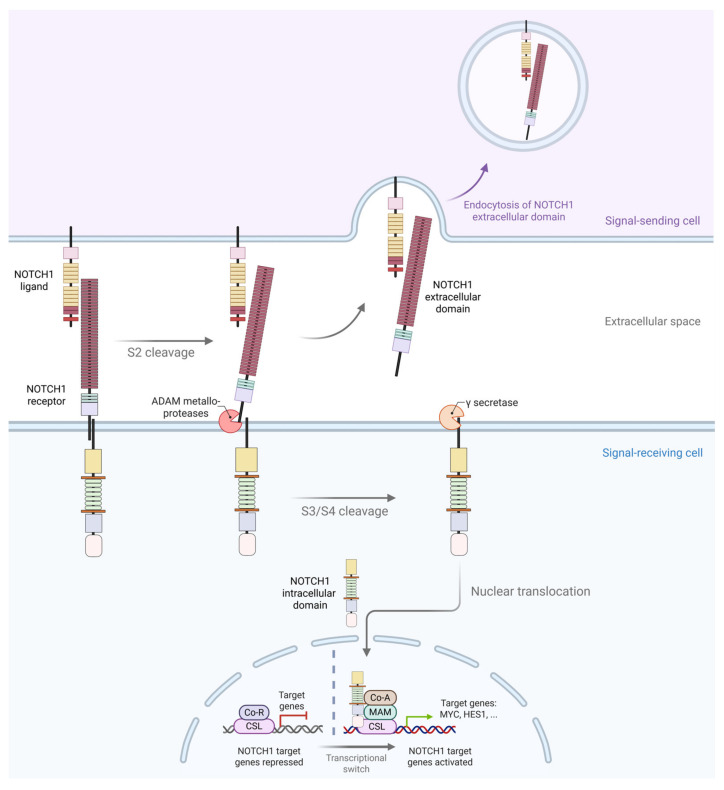
The canonical *NOTCH1* signaling pathway. Ligand binding induces a conformational change in the NOTCH1 receptor, exposing the S2 cleavage site within the negative regulatory region (NRR). ADAM family metalloproteases cleave at S2, releasing the extracellular domain, which is internalized by the ligand-presenting cell. The remaining membrane-tethered fragment undergoes cleavage at S3/S4 by the γ-secretase complex, liberating the *NOTCH* intracellular domain. The intracellular domain translocates to the nucleus, where it associates with CSL/RBP-Jκ and coactivators to regulate the transcription of target genes. Created with BioRender.com (https://biorender.com, accessed on 23 December 2025).

*NOTCH* signaling is a highly conserved cell–cell communication pathway that requires direct contact between a *NOTCH* receptor-expressing cell and a ligand-presenting cell [28,29,30]. Upon ligand binding, the NOTCH receptor undergoes a series of proteolytical cleavages (Figure 2) [28,29]. Ligand engagement induces a conformational change that exposes the S2 cleavage site, located 12 amino acids upstream of the transmembrane domain within the NRR [28,29,30,31]. This allows ADAM family metalloproteases to cleave the receptor at S2, resulting in the shedding of the NOTCH extracellular domain, which is then internalized by the ligand-presenting cell via endocytosis [28,29,30,31]. The remaining membrane-tethered fragment is subsequently processed at the S3 and S4 sites, located in the transmembrane domain, by the γ-secretase complex, releasing the intracellular domain, NICD (Figure 2) [28,29,30,31]. The NICD contains two nuclear localization signals that facilitate its translocation into the nucleus, where it associates with the CSL family of DNA-binding proteins via its RAM domain and Ankyrin repeats [28,29,31]. This interaction displaces histone deacetylases and converts CSL from a transcriptional repressor into an activator [28,29,31]. The NICD-CSL complex recruits chromatin-remodeling enzymes, such as histone acetyltransferases, leading to the transcriptional activation of canonical *NOTCH1* target genes, including *c-MYC* and *HES1* [30,31].

### 3.3. NOTCH1 in T-Cell Development and T-Cell Malignancies

**Figure 3:**
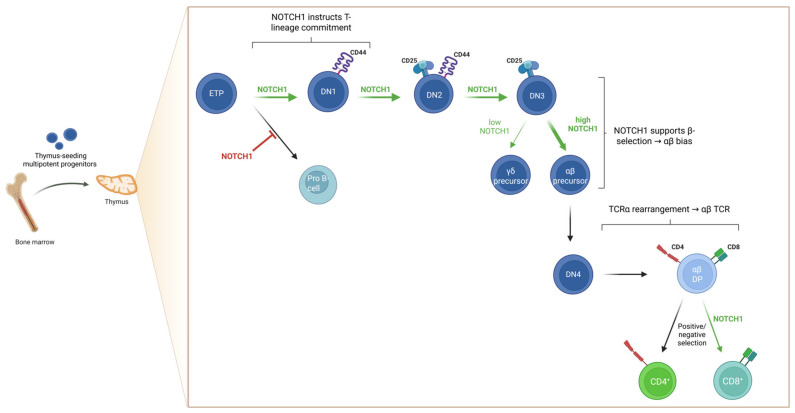
A schematic representation of normal T-cell development in the thymus, highlighting the stage-specific role of *NOTCH1* signaling. *NOTCH1* activity is essential for early T-lineage commitment in double-negative (DN) thymocytes, promotes progression through DN stages, and influences β-selection at the double-positive (DP) stage, ultimately supporting maturation into single-positive (SP) CD4^+^ and CD8^+^ T-cells. ETP, early T-cell progenitor, DN1, double-negative 1 stage; DN2, double-negative 2 stage; DN3, double-negative 3 stage; DN4, double-negative 4 stage. Created with BioRender.com (https://biorender.com, accessed on 23 December 2025).

*NOTCH* signaling plays a crucial role in hematopoiesis by regulating hematopoietic stem cell maintenance, self-renewal, and lineage commitment. NOTCH receptors are expressed throughout hematopoietic development and are activated via direct interactions with ligand-expressing bone marrow stromal or hematopoietic cells [27,31]. Among the NOTCH family, *NOTCH1* plays a particularly pivotal role in the lymphoid lineage, guiding T-cell lineage commitment and supporting multiple stages of thymocyte differentiation (Figure 3) [28,30]. Thymus-seeding progenitors are still uncommitted lymphoid progenitors that retain multipotency and require *NOTCH1*-*DLL4* signaling from the thymic epithelium to initiate the T-cell developmental program [27,28,33]. In the absence of *NOTCH1* activity, early T-cell progenitors (ETPs) fail to initiate the T-cell developmental program and instead adopt a B-cell fate within the thymic environment, underscoring *NOTCH1’s* instructive and lineage-determining role [34,35]. In contrast, continuously active *NOTCH1* in precursor cells enhance T-cell development while blocking B-cell development [36]. Beyond its involvement in T-cell lineage commitment, *NOTCH1* also plays a role in the subsequent stages of thymocyte development, in line with its continuous expression throughout thymocyte maturation [36]. *NOTCH1* is essential for supporting the proliferation and survival of early thymic progenitors and for guiding their progression through double-negative stages [36,37]. Moreover, *NOTCH1* signaling is pivotal at the DN3 thymocyte stage for αβ versus γδ T-cell lineage commitment [36,38]. *NOTCH1* activity drives αβ T-cell development by supporting productive Vβ-DJβ rearrangement, whereas reduced or absent signaling impairs TCRβ rearrangement and shifts differentiation toward the γδ lineage [28,30,38,39]. Although *NOTCH1* is primarily essential for early T-cell lineage commitment, as evidenced by its downregulation at the double-positive stage, some studies suggest that *NOTCH1* signaling also influences CD4/CD8 lineage choice, with a bias towards promoting CD8 differentiation [36]. Together, these findings establish *NOTCH1* as a key determinant of T-cell identity and lineage progression within the thymus.

Alongside *NOTCH1*, *NOTCH3* is coexpressed in early thymocyte subsets, with maximal expression in DN thymocytes, declining as cells transition towards the DP stage [40,41]. This dynamic regulation indicates that *NOTCH3* plays a modulatory role in the early phases of thymocyte development, specifically in the DN-to-DP transition. However, *NOTCH1* remains the only NOTCH receptor that is strictly required for T-cell lineage commitment, as the functional inhibition of NOTCH3 using blocking antibodies in fetal thymus organ cultures, as well as genetic *NOTCH3* depletion in vivo, did not significantly impair thymocyte differentiation [40,42].

While tightly regulated *NOTCH1* signaling is essential for normal T-cell development, its dysregulation is directly linked to malignant transformation. The aberrant activation of *NOTCH1*, caused by activating mutations and translocations, drives the pathogenesis of T-ALL and T-LBL by promoting uncontrolled proliferation and blocking the differentiation of immature T-cells [30,31].

It is worth noting that *NOTCH3* has also been implicated in leukemic transformation in a small subset of T-ALL cases, in line with its modulatory role in early thymocyte development. Activating *NOTCH3* mutations, as well as *NOTCH3* overexpression, have been reported in T-ALL [43,44]. However, data on *NOTCH3* involvement in T-LBL are currently lacking, and whether similar mechanisms operate in this disease remains unresolved. This underscores an interesting direction for future studies.

## 4. Landscape of *NOTCH1* Genetic Alterations in T-LBL

### 4.1. NOTCH1 Mutations in Pediatric T-LBL

**Figure 4:**
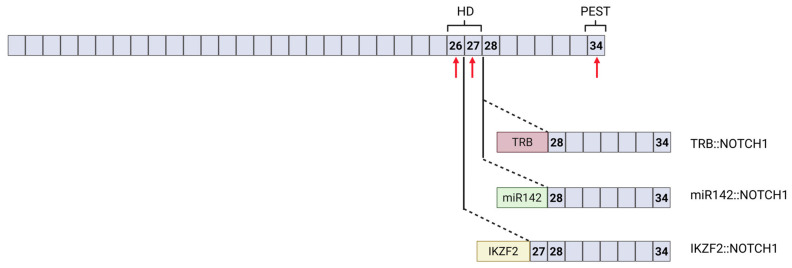
An exon map of the *NOTCH1* gene illustrating hotspot mutations in exons 26, 27, and 34 (red arrows) and translocation breakpoints. A schematic representation of the three resulting *NOTCH1* fusion genes is shown. HD, heterodimerization domain; PEST, proline–glutamate–serine–threonine-rich domain. Created with BioRender.com (https://biorender.com, accessed on 23 December 2025).

*NOTCH1* activating mutations are identified in over 50% of pediatric T-LBL patients, with a predominant localization to exons 26 and 27, encompassing the heterodimerization domain (HD), and exon 34, corresponding to the PEST regulatory domain (Figure 4) [30]. Mutations within the HD, most commonly single amino acid substitutions or small in-frame deletions and insertions, destabilize the receptor’s structure and weaken the interaction between the LNR and HD [30,45]. This structural disruption increases susceptibility to metalloprotease and γ-secretase cleavage, resulting in enhanced ligand hypersensitivity or ligand-independent activation through the constitutive release of the *NOTCH1* intracellular domain [30,45]. PEST domain mutations are typically frameshift or nonsense mutations that introduce a premature stop codon in the C-terminal region of *NOTCH1*, leading to the loss of the PEST degron [30]. This truncation impairs the ubiquitin-mediated degradation of the NOTCH1 intracellular domain (NICD) by the proteasome, thereby prolonging the NICD’s half-life and resulting in increased levels of activated NOTCH1 [30]. These two groups of mutations result in constitutive NOTCH1 activity, persistent expression of target genes such as *MYC* and *HES1*, and increased stability of the NICD, collectively driving leukemic cell survival and expansion [30,31].

### 4.2. NOTCH1 Fusions: Novel Rearrangements in Pediatric T-LBL

While *NOTCH1* mutations are well-established drivers of T-LBL, recent evidence highlights the emergence of *NOTCH1* gene fusions as another mechanism of aberrant *NOTCH1* signaling in this disease (Figure 4).

A case report by Yamamoto et al. described a *TRB*::*NOTCH1* fusion in a 41-year-old male T-LBL patient [46]. This translocation juxtaposes *NOTCH1* with the T-cell receptor β (TRB) locus, resulting in the overexpression of a truncated *NOTCH1* receptor under the control of *TRB* enhancer elements [46]. The resultant receptor lacks most of the extracellular domain, leading to the ligand-independent, constitutive activation of the *NOTCH1* signaling pathway. This finding provided the first evidence that *NOTCH1* fusions can promote T-cell transformation, paving the way for subsequent studies exploring their relevance in T-LBL pathogenesis [46].

Following this, te Vrugt et al. screened a large cohort of pediatric patients and found the *TRB*::*NOTCH1* fusion gene in 12 of 192 T-LBL cases (6%), while it was completely absent in the 167 pediatric T-ALL cases, highlighting this fusion gene as a specific molecular hallmark distinguishing T-LBL from T-ALL [20]. Importantly, patients with *TRB*::*NOTCH1* fusions had a significantly poorer prognosis, with event-free survival rates of 25% compared to 75–80% in fusion-negative patients, and an increased incidence of relapse [20]. Notably, none of the *TRB*::*NOTCH1*-positive cases harbored mutations in *NOTCH1* or *FBWX7*, further underscoring the distinct molecular profile associated with this fusion [20].

These findings are consistent with the initial report by Kroeze et al., identifying NOTCH1 fusions as a high-risk feature using RNA sequencing on diagnostic samples from 29 pediatric T-LBL patients and 39 pediatric T-ALL patients as a control cohort [15]. In this study, 6 of 29 T-LBL samples (21%) harbored a *NOTCH1* gene fusion, whereas none of the T-ALL samples showed such fusions, reinforcing the notion that *NOTCH1* rearrangements are specific to T-LBL. While the two previous studies focused exclusively on *TRB*::*NOTCH1* fusions, this study also identified, besides *TRB*, two other *NOTCH1* fusion partners: the microRNA host gene *miR142HG* and the transcription factor *IKZF2*. This study showed that all three fusion constructs produce active NICD, though through distinct mechanisms. The authors report that in both the *miR142HG*::*NOTCH1* and *TRBJ*::*NOTCH1* rearrangements, the translation of the fusion transcript initiates at an alternative internal methionine residue (Met1727) within exon 28 of *NOTCH1*, corresponding to the intracellular domain. According to this, these fusions bypass the extracellular and transmembrane regions that normally regulate receptor activation, making ligand binding and γ-secretase-mediated cleavage unnecessary. Consequently, this paper proposes that these two fusions constitutively activate *NOTCH1* signaling by mimicking a cleaved NICD. In contrast, the *IKZF2*::*NOTCH1* translocation joins the N-terminal DNA-binding domain of IKZF2 to the C-terminal intracellular portion of NOTCH1, generating a chimeric protein. The functional contribution of the IKZF2 domain remains uncharacterized but may potentially affect protein stability or subcellular localization or may combine IKZF2-DNA-binding activity with *NOTCH1* signaling activity. Further studies are required to elucidate its contribution. This study also offered further important insights into the molecular effects of *NOTCH1* translocations. For example, *NOTCH1* translocations induce much stronger transcriptional and signaling changes than *NOTCH1* mutations. Also, the *NOTCH1* signaling pathway is markedly more activated in fusion-positive T-LBL cases compared with those harboring wild-type or mutated *NOTCH1*, suggesting that the translocations exert a strong impact on T-LBL biology, reflected by the poor clinical outcomes.

### 4.3. Clinical Impact of NOTCH1 Alterations: Mutations vs. Fusions

From a clinical point of view, the presence of *NOTCH1* activating mutations is associated with a favorable prognosis. In a study by Callens et al., *NOTCH1*-mutated patients achieved a significantly higher 5-year EFS (96% ± 4%) compared with *NOTCH1* wild-type patients (53% ± 11%, *p* = 0.002) [47]. Similarly, Bonn et al. reported improved outcomes in *NOTCH1*-mutated cases, with 5-year pEFS values of 84% ± 5% versus 66% ± 7% (*p* = 0.021) [23].

In contrast, *NOTCH1* fusion-positive patients had markedly inferior outcomes [15]. Kroeze et al. reported that five of six *NOTCH1* fusion-positive patients experienced an event, four relapses during treatment and one therapy-related AML, versus only one event in the remaining cohort, yielding a significant difference in cumulative incidence (*p* < 0.001) [15]. Furthermore, Kroeze et al. reported that the presence of *NOTCH1* fusion genes was associated with elevated thymus and activation-regulated chemokine (CCL17/TARC) serum levels. CCL17 is an established diagnostic and prognostic marker in classical Hodgkin lymphoma, with validated roles in both disease detection and relapse surveillance [15,48,49]. The precise role of CCL17 in T-LBL pathogenesis remains to be elucidated, but this preliminary evidence suggests that elevated CCL17 levels could serve as a clinically relevant biomarker for identifying high-risk T-LBL patients at diagnosis.

### 4.4. The NOTCH1 Paradox

*NOTCH1* dysregulation in pediatric T-LBL presents a crucial prognostic paradox. While activating *NOTCH1* mutations in the HD and PEST domain are associated with a favorable outcome, translocations involving *NOTCH1* confer a dismal prognosis [15,23]. In both cases, the alteration results in a constitutively active NICD, driving the activation of downstream target genes. However, the clinical outcomes for patients differ markedly. A key distinction lies in the level and regulation of *NOTCH1* signaling; translocations typically place *NOTCH1* under the control of the promoter or enhancer elements of the fusion partners, leading to massive, unregulated expression, whereas mutated *NOTCH1* remains under its native promoter and exhibits more moderate activation. This difference likely contributes to the aggressive biology and therapy resistance observed in translocation-positive cases. Nevertheless, the precise mechanisms underlying this paradox remain to be fully elucidated.

## 5. Crosstalk with Other Molecular Pathways

**Figure 5:**
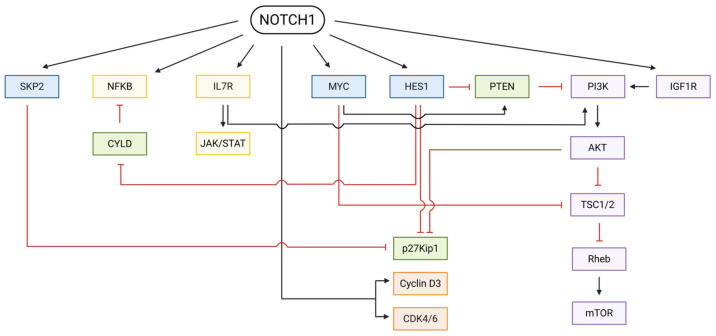
*NOTCH1*-regulated signaling landscape in T-ALL and T-LBL. Colored nodes represent functional groups of downstream pathways, including transcriptional targets (blue), cytokine/inflammatory signaling (yellow), tumor suppressors (green), cell cycle machinery (orange), and *PI3K*-*AKT*-*mTOR* survival signaling (purple). Black arrows indicate activation; red lines indicate inhibition, highlighting how *NOTCH1* integrates multiple pathways to promote proliferation, survival, and metabolic activity. Created with BioRender.com (https://biorender.com, accessed on 23 December 2025).

*NOTCH1* signaling profoundly rewires cellular programs by modulating many molecular pathways that control proliferation, survival, and lineage commitment. Among the altered pathways are the *PI3K*-*AKT*-*mTOR* pathway, *IL7*/*IL7R* signaling, the *JAK*/*STAT* pathway, the *NFκB* pathway, the *IGF1R* pathway and the modulation of cell cycle regulators (Figure 5).

First, *NOTCH1* activation strongly enhances *PI3K*-*AKT*-*mTOR* signaling through multiple complementary mechanisms that promote cellular growth, proliferation and survival while suppressing apoptosis. A central component to this regulation is the *NOTCH1*-mediated repression of *PTEN*, a key negative regulator of *PI3K* signaling [50,51,52]. *NOTCH1* induces the transcriptional repressor *HES1*, which binds the *PTEN* promoter and suppresses its expression, leading to increased PIP3 accumulation and enhanced AKT phosphorylation [50,51,52]. Conversely, *MYC*, another direct *NOTCH1* target, acts as a transcriptional activator of *PTEN* [50,52]. Thus, in *NOTCH1*-activated cells, *PTEN* expression is controlled by opposing inputs: repression by *HES1* and activation by *MYC* (Figure 5) [50,52]. However, the repressive effect of *HES1* dominates, resulting in an overall reduction in PTEN levels and elevated *PI3K*-*AKT* activity [50,52]. In addition to this classical *PTEN*-dependent route, *NOTCH1* also drives *mTOR* activation through a partially *PTEN*-independent mechanism [53]. As shown in T-ALL models, *NOTCH1* directly regulates the expression of *mTOR*-regulating components, sustaining *mTOR* activity even when *PI3K*-*AKT* input is limited [53]. This regulation occurs largely through *MYC*, which can, for example, inhibit the transcriptional repressor *TSC2*, thereby relieving the suppression of the *mTOR* pathway [54]. Thus, *NOTCH1* simultaneously amplifies *AKT* signaling and reinforces *mTOR* output through distinct but converging mechanisms, together driving proliferation, survival and metabolic fitness (Figure 5). Following this, the *PI3K*/*AKT* pathway is additionally activated through the *IGF1R* signaling axis, whose expression is transcriptionally upregulated by NICD binding to an intronic enhancer of the *IGF1R* gene [55].

*NOTCH1* activation also potentiates *IL-7*/*IL-7R* signaling, a pathway essential for thymocyte proliferation and survival (Figure 5) [56]. Upon ligand binding, the NICD interacts with a CSL-binding site in the *IL7R* promoter, directly inducing *IL7Rα* transcription [56]. The resulting increase in *IL7R* expression heightens cellular sensitivity to IL-7 and enhances downstream *PI3K*-*AKT* and *JAK*-*STAT* signaling [56,57]. So, the activation of the *IL7* pathway upon *NOTCH1* activation provides an additional layer of activation for the *PI3K*-*AKT* pathway. Also, the induction of *JAK1*, *JAK3* and *STAT5* upon *IL7R* stimulation further promotes cell growth and the proliferation of T-cells [58].

As noted earlier, *MYC* is a direct transcriptional target of *NOTCH1* [59,60]. The NICD/CSL complex binds the promoter sequences of *MYC* and increases *MYC* transcription [61]. This direct feed-forward loop regulation helps further explain the oncogenic effects of *NOTCH1* during leukemic transformation, as *MYC* drives cell cycle progression and proliferation, enhances protein synthesis, promotes ribosome biogenesis and modulates cellular metabolism [60,61].

Furthermore, *NOTCH1* signaling shows crosstalk with the *NFκB* pathway at different levels (Figure 5). Firstly, NICD is able to induce the *NFκB2* promoter [62,63]. Next, *HES1* can suppress the negative regulator *CYLD*, thereby enabling the activation of the *IKK* complex, a key downstream effector in the *NF-κB* pathway [63,64]. In addition, the NICD is also able to interact directly with active NFκB complexes in the nucleus, resulting in sustained *NFκB* signaling and the increased activation of *NFκB*-regulated genes [63,65].

Finally, *NOTCH1* signaling also plays a major role in regulating cell cycle progression (Figure 5). *HES1* represses the expression of the cell cycle inhibitor p27Kip1 by binding to its enhancer region [66]. In addition, *IL7R* signaling, which is upregulated by *NOTCH1*, indirectly suppresses p27Kip1 through PI3K activity [57]. *NOTCH1* also induces the transcription of *SKP2*, which promotes the proteasomal degradation of p27Kip1, facilitating premature entry into S-phase [67]. Moreover, *NOTCH1* enhances G1-S progression by upregulating cyclin D3 and CDK4/6 CSL-dependent transcription [59,68].

Overall, *NOTCH1* alterations orchestrate a broad network of signaling interactions, engaging pathways that regulate cell cycle progression and survival (Figure 5). This extensive crosstalk ultimately converges to sustain uncontrolled proliferation and resistance to cell death in malignant T-cells.

## 6. Therapy and Future Directions: Targeting NOTCH1

Given the high prevalence of *NOTCH1* alterations in T-cell malignancies and the substantial rate of relapsed T-LBL, the therapeutic targeting of the *NOTCH1* pathway has become a major focus in precision oncology and may offer a promising strategy to overcome relapsed or refractory cases driven by chemoresistance. Because *NOTCH1* is interconnected with numerous oncogenic networks that regulate proliferation, apoptosis, and drug sensitivity, multiple therapeutic strategies can be employed to downregulate the *NOTCH1* pathway [59]. Direct *NOTCH1*-targeting approaches include, among others, γ-secretase inhibitors, NOTCH1-specific monoclonal antibodies and anti-DLL4 antibodies [59]. More indirect inhibition can be achieved using agents, such as SERCA inhibitors or proteasome inhibitors, or by targeting key downstream effectors, such as *AKT*, *mTOR* or *NFκB* [59] (Table 2).

Over the past few years, γ-secretase inhibitors (GSIs) have attracted increasing interest as a therapeutic strategy for *NOTCH1*-altered T-ALL/T-LBL. As mentioned earlier, the canonical *NOTCH1* pathway requires cleavage by γ-secretase to release the NICD and to activate downstream target genes. GSIs are designed to block this cleavage and inhibit the activation of *NOTCH1*. At this point, preclinical in vitro and in vivo studies have shown the sensitivity of *NOTCH1*-mutated and *NOTCH1* fusion-positive T-ALL/T-LBL cells to several GSIs, evidenced by reduced proliferation and increased apoptosis [69,70,71,72]. However, inconsistent reports on clinical benefit and the occurrence of severe gastrointestinal toxicity preclude the implementation of these agents in front-line treatment protocols [73,74]. Combination strategies using lower doses of GSIs with other agents may enhance anti-tumor activity while potentially mitigating GSI-related toxicity. For example, an in vivo preclinical study showed a decrease in GSI-induced gut toxicity and an increased anti-leukemic effect when mice were treated with a combination of GSIs and dexamethasone [74]. Another study evaluated the combination therapy of GSIs and the mTOR inhibitor rapamycin in T-ALL mouse models, reporting reduced leukemic growth and increased overall survival [75]. Clinical trials of GSIs combined with steroids in T-ALL/T-LBL were generally safe but showed minimal clinical benefit due to low response rates, indicating the need for more effective strategies [76,77]. Another strategy to inhibit γ-secretase activity while minimizing toxicity is the use of selective GSIs, targeting a specific γ-secretase complex. The selective inhibitor MRK-560 specifically targets PSEN1, the presenilin-1 catalytic subunit of one γ-secretase complex, leading to decreased NICD production and the reduced proliferation of *NOTCH1*-dependent T-ALL cells [73,78]. In T-ALL xenograft mouse models, MRK-560 treatment improved survival without inducing gastrointestinal toxicity typically associated with broad γ-secretase inhibition [73,78]. Moreover, MRK-560 enhanced the sensitivity of T-ALL cells to dexamethasone, demonstrating a synergistic therapeutic effect [73].

Alternatively, the use of monoclonal antibodies against NOTCH1 or its ligand DLL4 has been explored. OMP-52M51 (Brontictuzumab), an anti-NOTCH1 mAb that binds the negative regulatory region, increased apoptosis and reduced the proliferation of T-ALL cells in vivo [79]. Additionally, the combination therapy of OMP-52M51 and dexamethasone enhanced therapeutic efficacy significantly in T-ALL PDX mouse models [79]. The drug was evaluated in a Phase 1 clinical trial involving 24 patients with relapsed or refractory hematologic malignancies and showed good tolerability; however, only moderate anti-tumor activity was observed, as only one partial response and two cases with stable disease were reported [80]. Antibodies targeting DLL4 have shown promising preclinical efficacy in T-cell malignancies. For example, the DLL4-targeted antibody OMP-21R30 impaired *NOTCH1* signaling and the growth of T-ALL cells in vivo [81]. Different anti-DLL4 monoclonal antibodies underwent evaluation in Phase 1 dose escalation trials involving patients with advanced solid malignancies (including colorectal cancer, pancreatic cancer, sarcomas, breast cancer…), showing an acceptable safety profile and measurable signs of anti-tumor efficacy [82,83]. No trials exploring the use of anti-DLL4 antibodies in T-cell malignancies have been conducted yet.

*NOTCH1* targeting has also been explored through the small molecule inhibition of the sarco/endoplasmic reticulum calcium ATPase (SERCA). SERCA plays an important role in the early maturation and proper folding of NOTCH1 within the endoplasmic reticulum [84]. Inhibiting SERCA prevents NOTCH1 from reaching the cell surface, thereby blocking downstream *NOTCH1* signaling [84]. Different SERCA inhibitors have been developed. Early work focused on thapsigargin and various thapsigargin analogs, which demonstrated potent activity against *NOTCH1*-driven T-ALL but showed limited clinical utility due to dose-limiting cardiac and gastrointestinal toxicities in preclinical studies [84,85]. More recently, the SERCA inhibitor CAD204520 suppressed *NOTCH1*-mutated leukemic cells in a T-ALL xenograft model without causing cardiac toxicity [85].

Another indirect targeting approach involves the use of proteasome inhibitors. For example, Bortezomib, a proteasome inhibitor approved for clinical use in multiple myeloma, has been shown to effectively inhibit *NOTCH1* signaling in T-ALL by reducing NF-κB activity and downregulating downstream effectors such as *MYC* and *Pre-Tα* [86,87]. This resulted in suppressed T-ALL cell growth both in vitro and in vivo, with a strong synergistic effect when combined with dexamethasone [86,87]. Although Bortezomib has been tested in clinical trials for T-ALL, both in re-induction settings and as part of first-line treatment with high efficacy, these studies were not designed specifically for *NOTCH1*-driven disease [88,89]. Nevertheless, the preclinical evidence indicates that proteasome inhibition represents a promising, albeit indirect, strategy to target *NOTCH1* signaling.

Beyond preventing *NOTCH1* activation at the receptor or cleavage level, efforts have also focused on inhibiting the downstream transcriptional machinery that executes *NOTCH1*-dependent gene expression. For example, SAHM1 is a stapled peptide derived from the coactivator MAML1, which normally bridges NICD and CSL to form the NOTCH transcriptional activation complex. SAHM1 competes for this connection by binding NICD and CSL, thereby preventing MAML1 recruitment and blocking the assembly of the active NOTCH complex [90]. In *NOTCH1*-dependent T-ALL cell lines, SAHM1 treatment reduces cellular proliferation by suppressing the expression of the target genes without affecting NICD protein stability. In vivo, the administration of SAHM1 in a murine *NOTCH1*-driven T-ALL model lowers leukemic burden and decreases leukemic infiltration. Similarly, IMR-1 is a small molecule inhibitor that blocks MAML1 recruitment to chromatin, thereby shutting down *NOTCH1*-dependent transcription [91]. Although it has not been evaluated in T-ALL or T-LBL, preclinical studies in *NOTCH1*-dependent esophageal adenocarcinoma demonstrated reduced target gene expression and significant tumor inhibition without detectable toxicity in patient-derived xenograft models [91]. Additionally, CB-103 is a small molecule inhibitor that blocks the assembly of the NICD-CSL complex, suppressing *NOTCH*-driven gene expression, inhibiting T-ALL cell line growth, and prolonging survival in *NOTCH1*-mutated T-ALL patient-derived xenograft models [92]. A recent case report also described a complete clinical response in a 24-year-old patient with relapsed high-risk T-ALL treated with CB-103, underscoring its potential as a promising therapeutic approach [93]. Complementing these strategies, the RBPJ Inhibitor-1, RIN1, acts even further downstream by inhibiting the DNA-bound effector of the NICD-CSL-MAML complex, RBPJ, leading to the reduced proliferation of *NOTCH1*-dependent T-ALL cell lines [94]. Also, the inhibition of key downstream effectors such as mTOR has been explored. Indeed, rapamycin, an mTOR inhibitor, has been shown to effectively inhibit T-ALL cell growth in vitro [75].

An important emerging consideration in evaluating the therapeutic potential of targeting the *NOTCH1* pathway in T-LBL is the specific *NOTCH1* alteration. Specifically, the precise breakpoint of *NOTCH1* translocations determines the structure and function of the resulting fusion protein and consequently influences the sensitivity to GSIs. Two distinct types of *TRB*::*NOTCH1* fusion proteins have been described. Yamamoto et al. reported a fusion that produces a membrane-bound NOTCH1 protein lacking the extracellular domain but retaining the transmembrane domain, including the γ-secretase cleavage site [46]. This form remains dependent on γ-secretase for activation [46]. In contrast, Kroeze et al. described a fusion that results in a cytoplasmic NOTCH1 protein, which is constitutively active and independent of γ-secretase cleavage [15]. This distinction has functional consequences, as evidenced by in vitro studies. The CUTLL1 cell line, which encodes a membrane-bound TRB::NOTCH1 fusion protein, was sensitive to GSIs [70]. The SUPT1 cell line, which also has a TRB::NOTCH1 translocation but produces a cytoplasmic form, was resistant to GSIs. Similarly, studies have shown that the SERCA inhibitor thapsigargin does not block NICD production in the SUPT1 cell line, whereas it does inhibit NICD generation in cell lines that carry *NOTCH1* alleles with heterodimerization domain mutations [84]. These observations thus exemplify how drug sensitivity in T-LBL is highly dependent on the exact breakpoint and resulting structure of the NOTCH1 fusion protein. These observations thus strongly indicate the need for a personalized treatment approach for T-LBL patients harboring *NOTCH1* alterations, rather than a one-size-fits-all solution.

Several additional strategies remain to be explored. For instance, *NOTCH1*-driven T-cell malignancies might be indirectly suppressed by inhibiting the *RAS*-*MAPK* pathway (e.g., with MEK inhibitors), counteracting *PTEN* inhibition, or modulating cell cycle regulators such as *CDKN2A* and *CDKN2B*. Other promising directions include the exploration of ADAM inhibitors, which block the S2 cleavage step, mediated by ADAM metalloproteases, upstream of γ-secretase, and thereby prevent the activation of the NOTCH receptor [28,29,30,31]. To identify effective combinations and novel targets, large-scale drug screening efforts will be essential.

**Table 2:** Overview of pharmacological strategies targeting *NOTCH1* signaling in T-cell acute lymphoblastic leukemia preclinical models.

Compound	Type of Inhibitor	Experimental Model	Key Findings	Ref.
Compound E	Gamma secretase inhibitor	CUTLL1 cell line	Reduced levels of NICD. Impaired proliferation due to cell cycle arrest and increased apoptosis.	[70]
MRK-003	Gamma secretase inhibitor	T-ALL cell lines (DND-41, HPB-ALL, TALL-1) T-ALL xenograft models	Decrease in metabolic activity. Decreased proliferation and cell viability due to cell cycle arrest and apoptosis. Apoptosis of leukemia cells. Anti-tumor efficacy.	[71,72]
MRK-560	Selective gamma secretase inhibitor	T-ALL cell lines (HPB-ALL, DND-41, SUPT1) T-ALL xenograft models and PDX models	Impaired leukemia progression and prolonged survival. Reduced toxicity. Synergy with dexamethasone.	[73,78]
XXI	Gamma secretase inhibitor	Primary T-ALL cell cultures	Downregulation of NICD.	[69]
OMP-52M51	*NOTCH1* monoclonal antibody	T-ALL patient-derived xenograft models	Increased apoptosis and reduced proliferation of T-ALL cells. Treatment increased survival of mice. Synergistic effect with dexamethasone.	[79]
OMP-21R30	DLL4 antibody	T-ALL patient-derived xenograft models	Delayed leukemia growth and increased apoptosis.	[81]
Thapsigargin	SERCA inhibitor	T-ALL cell lines (DND42, MOLT4) and MOLT4 xenograft models	Inhibition of *NOTCH1* and cell cycle arrest in T-ALL cell lines. Inhibition of tumor growth in treated mice compared to vehicle. High toxicity.	[84]
CAD204520	SERCA inhibitor	In vitro and in vivo *NOTCH1*-mutated T-ALL models	Reduced levels of mature NOTCH1. Lower cell viability and decreased cell growth of T-ALL cells.	[85]
Bortezomib	Proteasome inhibitor	T-ALL cell lines (JURKAT, MOLT4, CEM) and MOLT4 xenograft models	Bortezomib inhibits *NOTCH1* activity and induces apoptosis. Inhibition of tumor growth, increase in overall survival. Strong synergistic effect with dexamethasone.	[86,87]
SAHM1	Hydrocarbon-stapled α-helical peptide	Panel of human T-ALL cell lines (KOPT-K1, JURKAT, SUPT1, MOLT-4…)	Suppressed expression of *NOTCH1* target genes. Reduced proliferation of cells.	[90]
CB-103	Small molecule PPI	Panel of human T-ALL cell lines (KOPT-K1, DND-41, TALL-1, HPB-ALL, RPMI-8402) T-ALL patient-derived xenograft model	Induction of cell cycle arrest and apoptosis and impaired proliferation of T-ALL cell lines. Anti-tumor activity in xenograft models.	[92]
RIN1	RBPJ inhibitor-1	*NOTCH1*-dependent hematological cell lines (JURKAT, KOPT-K1, REC-1)	Decreased proliferation of cell lines.	[94]

## 7. Conclusions

A major challenge in T-LBL is the poor knowledge about its molecular pathobiology. *NOTCH1* alterations represent the most frequent genetic abnormalities in pediatric T-ALL and T-LBL. Activating mutations and chromosomal translocations hyperactivate the *NOTCH1* signaling pathway, driving the malignant transformation of immature T-cells and disrupting normal T-cell development [30,31]. This aberrant signaling rewires key regulatory networks and pro-survival pathways, including *PI3K*-*AKT*, *NF*-*κB*, and *IL7R*, promoting the uncontrolled proliferation and survival of leukemic cells. While *NOTCH1* mutations occur in both T-ALL and T-LBL and are generally associated with a favorable prognosis, *NOTCH1* translocations are largely specific to T-LBL and correlate with a poor prognosis. The studies by Yamamoto et al., te Vrugt et al., and Kroeze et al. lay a solid foundation for further investigating *NOTCH1* fusions in T-LBL. *NOTCH1* fusions may help identify high-risk patients who would benefit from intensified or alternative treatment strategies, avoiding relapse, which is associated with very poor prognoses.

Therapeutic strategies targeting the *NOTCH1* pathway are being investigated, but their development is mostly stuck at the preclinical stage, as clinical application is limited by toxicity, highlighting the need for further research. Importantly, most efforts to target *NOTCH1* signaling have focused on T-ALL models, with only limited data available for T-LBL specifically. Nevertheless, these insights may remain highly relevant to T-LBL given the shared dependence on aberrant *NOTCH1* activity. Therapeutic vulnerabilities identified in T-ALL are likely to translate to T-LBL biology. However, dedicated investigations using T-LBL models are essential to confirm whether these findings can be fully extrapolated. Moreover, the identification of T-LBL-specific *NOTCH1* fusion events underscores the need for research tailored to this disease context. Considering that functionally different *NOTCH1* alterations are present in patients, specific *NOTCH1* alteration-dependent therapies might be needed, rather than the possibility of one general *NOTCH1*-targeted treatment approach.

In conclusion, a T-LBL-specific research strategy is urgently needed, given the biological and clinical differences from its leukemic counterpart, T-ALL. Such an approach will be crucial to unravel the unique pathobiology of this disease and identify vulnerabilities that can be exploited therapeutically. Interestingly, research into the recently discovered *NOTCH1* fusion genes in the context of T-LBL represents a crucial challenge in understanding T-LBL-specific drivers and developing precision medicine strategies that overcome chemoresistance and reduce the high relapse rates, ultimately improving outcomes for affected children.

## Data Availability

No new data were created or analyzed in this study. Data sharing is not applicable to this article.

## References

[1] Temple W.C., Mueller S., Hermiston M.L., Burkhardt B. (2023). Diagnosis and management of lymphoblastic lymphoma in children, adolescents and young adults. Best. Pract. Res. Clin. Haematol..

[2] Burkhardt B., Hermiston M.L. (2019). Lymphoblastic lymphoma in children and adolescents: Review of current challenges and future opportunities. Br. J. Haematol..

[3] Choi J.K., Quintanilla-Martinez L. (2025). Pediatric lymphomas: Overview and diagnostic challenges. Virchows Arch..

[4] Burkhardt B., Mueller S., Khanam T., Perkins S.L. (2016). Current status and future directions of T-lymphoblastic lymphoma in children and adolescents. Br. J. Haematol..

[5] Burkhardt B., Zimmermann M., Oschlies I., Niggli F., Mann G., Parwaresch R., Riehm H., Schrappe M., Reiter A. (2005). The impact of age and gender on biology, clinical features and treatment outcome of non-Hodgkin lymphoma in childhood and adolescence. Br. J. Haematol..

[6] Te Vrugt M., Newman H., Teachey D.T., Burkhardt B. (2025). T-cell lymphoblastic lymphoma in children, adolescents and young adults. Br. J. Haematol..

[7] Poirel H.A., Ambrosio M.R., Piccaluga P.P., Leoncini L., Lenz G., Salles G. (2019). Pathology and Molecular Pathogenesis of Burkitt Lymphoma and Lymphoblastic Lymphoma. Aggressive Lymphomas.

[8] Rosolen A., Perkins S.L., Pinkerton C.R., Guillerman R.P., Sandlund J.T., Patte C., Reiter A., Cairo M.S. (2015). Revised International Pediatric Non-Hodgkin Lymphoma Staging System. J. Clin. Oncol..

[9] Murphy S.B. (1980). Classification, staging and end results of treatment of childhood non-Hodgkin’s lymphomas: Dissimilarities from lymphomas in adults. Semin. Oncol..

[10] Attarbaschi A., Beishuizen A., Mann G., Rosolen A., Burkhardt B. (2021). European Standard Clinical Practice Recommendations for Non-Hodgkin Lymphoma of Childhood and Adolescence.

[11] Kroeze E., Loeffen J.L.C., Poort V.M., Meijerink J.P.P. (2020). T-cell lymphoblastic lymphoma and leukemia: Different diseases from a common premalignant progenitor?. Blood Adv..

[12] WHO Classification of Tumours Editorial Board (2024). WHO Classification of Tumours: Haematolymphoid Tumours.

[13] Harris N.L., Jaffe E.S., Stein H., Banks P.M., Chan J.K.C., Cleary M.L., Delsol G., De Wolf-Peeters C., Falini B., Gatter K.C. (1994). A Revised European-American Classification of Lymphoid Neoplasms: A Proposal from the International Lymphoma Study Group. Blood.

[14] Raetz E.A., Perkins S.L., Bhojwani D., Smock K., Philip M., Carroll W.L., Min D.-J. (2006). Gene expression profiling reveals intrinsic differences between T-cell acute lymphoblastic leukemia and T-cell lymphoblastic lymphoma. Pediatr. Blood Cancer.

[15] Kroeze E., Kleisman M.M., Kester L.A., Scheijde-Vermeulen M.A., Sonneveld E., Buijs-Gladdines J.G.C., Hagleitner M.M., Meyer-Wentrup F.A.G., Veening M.A., Beishuizen A. (2024). NOTCH1 fusions in pediatric T-cell lymphoblastic lymphoma: A high-risk subgroup with CCL17 (TARC) levels as diagnostic biomarker. Hemasphere.

[16] Reiter A., Schrappe M., Ludwig W.-D., Tiemann M., Parwaresch R., Zimmermann M., Schirg E., Henze G., Schellong G., Gadner H. (2000). Intensive ALL-type therapy without local radiotherapy provides a 90% event-free survival for children with T-cell lymphoblastic lymphoma: A BFM Group report. Blood.

[17] Landmann E., Burkhardt B., Zimmermann M., Meyer U., Woessmann W., Klapper W., Wrobel G., Rosolen A., Pillon M., Escherich G. (2017). Results and conclusions of the European Intergroup EURO-LB02 trial in children and adolescents with lymphoblastic lymphoma. Haematologica.

[18] Burkhardt B., Reiter A., Landmann E., Lang P., Lassay L., Dickerhoff R., Lakomek M., Henze G., Stackelberg A.v. (2009). Poor Outcome for Children and Adolescents with Progressive Disease or Relapse of Lymphoblastic Lymphoma: A Report from the Berlin-Frankfurt-Muenster Group. J. Clin. Oncol..

[19] Si Lim S.J., Ford J.B., Hermiston M.L. (2023). How I treat newly diagnosed and refractory T-cell acute lymphoblastic lymphoma in children and young adults. Blood.

[20] Te Vrugt M., Wessolowski J., Randau G., Alfert A., Mueller S., Scholten K., Sopalla C., Lanvers-Kaminsky C., Hotfilder M., Lamp F. (2024). Pediatric T-cell lymphoblastic lymphomas but not leukemias harbor TRB::NOTCH1 fusions with unfavorable outcome. Blood.

[21] Bontoux C., Simonin M., Garnier N., Lhermitte L., Touzart A., Andrieu G., Bruneau J., Lengliné E., Plesa A., Boissel N. (2022). Oncogenetic landscape of T-cell lymphoblastic lymphomas compared to T-cell acute lymphoblastic leukemia. Mod. Pathol..

[22] Khanam T., Sandmann S., Seggewiss J., Ruether C., Zimmermann M., Norvil A.B., Bartenhagen C., Randau G., Mueller S., Herbrueggen H. (2021). Integrative genomic analysis of pediatric T-cell lymphoblastic lymphoma reveals candidates of clinical significance. Blood.

[23] Bonn B.R., Rohde M., Zimmermann M., Krieger D., Oschlies I., Niggli F., Wrobel G., Attarbaschi A., Escherich G., Klapper W. (2013). Incidence and prognostic relevance of genetic variations in T-cell lymphoblastic lymphoma in childhood and adolescence. Blood.

[24] Balbach S.T., Makarova O., Bonn B.R., Zimmermann M., Rohde M., Oschlies I., Klapper W., Rössig C., Burkhardt B. (2016). Proposal of a genetic classifier for risk group stratification in pediatric T-cell lymphoblastic lymphoma reveals differences from adult T-cell lymphoblastic leukemia. Leukemia.

[25] Veltri G., Silvestri C., Gallingani I., Sandei M., Vencato S., Lovisa F., Cortese G., Pillon M., Carraro E., Bresolin S. (2021). Ruxolitinib as a Novel Therapeutic Option for Poor Prognosis T-LBL Pediatric Patients. Cancers.

[26] Kroeze E., Kleisman M.M., Hagelaar R., Bladergroen R.S., Kester L.A., Scheijde-Vermeulen M.A., van Dijk F., Meijerink J.P.P., Kuiper R.P., Loeffen J.L.C. (2024). T-cell lymphoblastic lymphoma compared with T-cell acute lymphoblastic leukemia: Similar subtypes and different fusions. Blood Neoplasia.

[27] Stier S., Cheng T., Dombkowski D., Carlesso N., Scadden D.T. (2002). Notch1 activation increases hematopoietic stem cell self-renewal in vivo and favors lymphoid over myeloid lineage outcome. Blood.

[28] Li X., von Boehmer H. (2011). Notch Signaling in T-Cell Development and T-ALL. ISRN Hematol..

[29] Kopan R., Ilagan M.X.G. (2009). The Canonical Notch Signaling Pathway: Unfolding the Activation Mechanism. Cell.

[30] Ferrando A.A. (2009). The role of NOTCH1 signaling in T-ALL. Hematology.

[31] Suresh S., Irvine A.E. (2015). The NOTCH signaling pathway in normal and malignant blood cell production. J. Cell Commun. Signal..

[32] Deregowski V., Gazzerro E., Priest L., Rydziel S., Canalis E. (2009). Role of the Ram Domain and Ankyrin Repeats on Notch Signaling and Activity in Cells of Osteoblastic Lineage. J. Bone Miner. Res..

[33] Deftos M.L., Bevan M.J. (2000). Notch signaling in T cell development. Curr. Opin. Immunol..

[34] Wilson A., MacDonald H.R., Radtke F. (2001). Notch 1–Deficient Common Lymphoid Precursors Adopt a B Cell Fate in the Thymus. J. Exp. Med..

[35] Radtke F., Wilson A., Stark G., Bauer M., van Meerwijk J., MacDonald H.R., Aguet M. (1999). Deficient T cell fate specification in mice with an induced inactivation of Notch1. Immunity.

[36] Rothenberg E.V., Taghon T. (2005). Molecular genetics of T cell development. Annu. Rev. Immunol..

[37] Schmitt T.M., Ciofani M., Petrie H.T., Zúñiga-Pflücker J.C. (2004). Maintenance of T cell specification and differentiation requires recurrent notch receptor-ligand interactions. J. Exp. Med..

[38] Washburn T., Schweighoffer E., Gridley T., Chang D., Fowlkes B.J., Cado D., Robey E. (1997). Notch Activity Influences the αβ versus γδ T Cell Lineage Decision. Cell.

[39] Wolfer A., Wilson A., Nemir M., MacDonald H.R., Radtke F. (2002). Inactivation of Notch1 impairs VDJbeta rearrangement and allows pre-TCR-independent survival of early alpha beta Lineage Thymocytes. Immunity.

[40] Shi J., Fallahi M., Luo J.-L., Petrie H.T. (2011). Nonoverlapping functions for Notch1 and Notch3 during murine steady-state thymic lymphopoiesis. Blood.

[41] Bellavia D., Campese A.F., Vacca A., Gulino A., Screpanti I. (2003). Notch3, another Notch in T cell development. Semin. Immunol..

[42] Waegemans E., Van de Walle I., De Medts J., De Smedt M., Kerre T., Vandekerckhove B., Leclercq G., Wang T., Plum J., Taghon T. (2014). Notch3 activation is sufficient but not required for inducing human T-lineage specification. J. Immunol..

[43] Bernasconi-Elias P., Hu T., Jenkins D., Firestone B., Gans S., Kurth E., Capodieci P., Deplazes-Lauber J., Petropoulos K., Thiel P. (2016). Characterization of activating mutations of NOTCH3 in T-cell acute lymphoblastic leukemia and anti-leukemic activity of NOTCH3 inhibitory antibodies. Oncogene.

[44] Noura M., Yasuda T., Kiyoi H., Hayakawa F. (2025). Induction of T-Cell Differentiation by KLF4 in T-Cell Acute Lymphoblastic Leukemia Cells Harboring Activating Mutation in NOTCH3. FASEB J..

[45] Malecki M.J., Sanchez-Irizarry C., Mitchell J.L., Histen G., Xu M.L., Aster J.C., Blacklow S.C. (2006). Leukemia-associated mutations within the NOTCH1 heterodimerization domain fall into at least two distinct mechanistic classes. Mol. Cell Biol..

[46] Yamamoto K., Nakamachi Y., Yakushijin K., Miyata Y., Okamura A., Kawano S., Matsuoka H., Minami H. (2013). A novel TRB@/NOTCH1 fusion gene in T-cell lymphoblastic lymphoma with t(7;9)(q34;q34). Eur. J. Haematol..

[47] Callens C., Baleydier F., Lengline E., Abdelali R.B., Petit A., Villarese P., Cieslak A., Minard-Colin V., Rullier A., Moreau A. (2012). Clinical impact of NOTCH1 and/or FBXW7 mutations, FLASH deletion, and TCR status in pediatric T-cell lymphoblastic lymphoma. J. Clin. Oncol..

[48] Teesink S.A., Visser L., Nijland M., Keijzer K., Niezink A.G.H., Kroesen B.-J., van den Berg A., Diepstra A., Plattel W.J. (2025). Serum TARC: A biomarker for early detection or exclusion of relapse in classic Hodgkin lymphoma. Blood Adv..

[49] Weihrauch M.R., Manzke O., Beyer M., Haverkamp H., Diehl V., Bohlen H., Wolf J., Schultze J.L. (2005). Elevated serum levels of CC thymus and activation-related chemokine (TARC) in primary Hodgkin’s disease: Potential for a prognostic factor. Cancer Res..

[50] Palomero T., Dominguez M., Ferrando A.A. (2008). The role of the PTEN/AKT Pathway in NOTCH1-induced leukemia. Cell Cycle.

[51] Wong G.W., Knowles G.C., Mak T.W., Ferrando A.A., Zúñiga-Pflücker J.C. (2012). HES1 opposes a PTEN-dependent check on survival, differentiation, and proliferation of TCRβ-selected mouse thymocytes. Blood.

[52] Palomero T., Sulis M.L., Cortina M., Real P.J., Barnes K., Ciofani M., Caparros E., Buteau J., Brown K., Perkins S.L. (2007). Mutational loss of PTEN induces resistance to NOTCH1 inhibition in T-cell leukemia. Nat. Med..

[53] Chan S.M., Weng A.P., Tibshirani R., Aster J.C., Utz P.J. (2007). Notch signals positively regulate activity of the mTOR pathway in T-cell acute lymphoblastic leukemia. Blood.

[54] Hales E.C., Taub J.W., Matherly L.H. (2014). New insights into Notch1 regulation of the PI3K–AKT–mTOR1 signaling axis: Targeted therapy of γ-secretase inhibitor resistant T-cell acute lymphoblastic leukemia. Cell. Signal..

[55] Medyouf H., Gusscott S., Wang H., Tseng J.-C., Wai C., Nemirovsky O., Trumpp A., Pflumio F., Carboni J., Gottardis M. (2011). High-level IGF1R expression is required for leukemia-initiating cell activity in T-ALL and is supported by Notch signaling. J. Exp. Med..

[56] González-García S., García-Peydró M., Martín-Gayo E., Ballestar E., Esteller M., Bornstein R., de la Pompa J.L., Ferrando A.A., Toribio M.L. (2009). CSL–MAML-dependent Notch1 signaling controls T lineage–specific IL-7Rα gene expression in early human thymopoiesis and leukemia. J. Exp. Med..

[57] Barata J.T., Silva A., Brandao J.G., Nadler L.M., Cardoso A.A., Boussiotis V.A. (2004). Activation of PI3K Is Indispensable for Interleukin 7–mediated Viability, Proliferation, Glucose Use, and Growth of T Cell Acute Lymphoblastic Leukemia Cells. J. Exp. Med..

[58] Foxwell B.M.J., Beadling C., Guschin D., Kerr I., Cantrell D. (1995). Interleukin-7 can induce the activation of Jak 1, Jak 3 and STAT 5 proteins in murine T cells. Eur. J. Immunol..

[59] Gharaibeh L., Elmadany N., Alwosaibai K., Alshaer W. (2020). Notch1 in Cancer Therapy: Possible Clinical Implications and Challenges. Mol. Pharmacol..

[60] Weng A.P., Millholland J.M., Yashiro-Ohtani Y., Arcangeli M.L., Lau A., Wai C., Del Bianco C., Rodriguez C.G., Sai H., Tobias J. (2006). c-Myc is an important direct target of Notch1 in T-cell acute lymphoblastic leukemia/lymphoma. Genes. Dev..

[61] Palomero T., Lim W.K., Odom D.T., Sulis M.L., Real P.J., Margolin A., Barnes K.C., O’Neil J., Neuberg D., Weng A.P. (2006). NOTCH1 directly regulates c-MYC and activates a feed-forward-loop transcriptional network promoting leukemic cell growth. Proc. Natl. Acad. Sci. USA.

[62] Oswald F., Liptay S., Adler G., Schmid R.M. (1998). NF-κB2 Is a Putative Target Gene of Activated Notch-1 via RBP-Jκ. Mol. Cell. Biol..

[63] Hoofd C., Giambra V., Weng A.P., Miele L., Artavanis-Tsakonas S. (2018). Notch Signaling in T-Cell Acute Lymphoblastic Leukemia and Other Hematologic Malignancies. Targeting Notch in Cancer: From the Fruit Fly to the Clinic.

[64] Espinosa L., Cathelin S., D’Altri T., Trimarchi T., Statnikov A., Guiu J., Rodilla V., Inglés-Esteve J., Nomdedeu J., Bellosillo B. (2010). The Notch/Hes1 Pathway Sustains NF-κB Activation through CYLD Repression in T Cell Leukemia. Cancer Cell.

[65] Shin H.M., Minter L.M., Cho O.H., Gottipati S., Fauq A.H., Golde T.E., Sonenshein G.E., Osborne B.A. (2006). Notch1 augments NF-κB activity by facilitating its nuclear retention. EMBO J..

[66] Murata K., Hattori M., Hirai N., Shinozuka Y., Hirata H., Kageyama R., Sakai T., Minato N. (2005). Hes1 directly controls cell proliferation through the transcriptional repression of p27Kip1. Mol. Cell Biol..

[67] Dohda T., Maljukova A., Liu L., Heyman M., Grandér D., Brodin D., Sangfelt O., Lendahl U. (2007). Notch signaling induces SKP2 expression and promotes reduction of p27Kip1 in T-cell acute lymphoblastic leukemia cell lines. Exp. Cell Res..

[68] Joshi I., Minter L.M., Telfer J., Demarest R.M., Capobianco A.J., Aster J.C., Sicinski P., Fauq A., Golde T.E., Osborne B.A. (2009). Notch signaling mediates G1/S cell-cycle progression in T cells via cyclin D3 and its dependent kinases. Blood.

[69] Kindler T., Cornejo M.G., Scholl C., Liu J., Leeman D.S., Haydu J.E., Fröhling S., Lee B.H., Gilliland D.G. (2008). K-RasG12D–induced T-cell lymphoblastic lymphoma/leukemias harbor Notch1 mutations and are sensitive to γ-secretase inhibitors. Blood.

[70] Palomero T., Barnes K.C., Real P.J., Glade Bender J.L., Sulis M.L., Murty V.V., Colovai A.I., Balbin M., Ferrando A.A. (2006). CUTLL1, a novel human T-cell lymphoma cell line with t(7;9) rearrangement, aberrant NOTCH1 activation and high sensitivity to γ-secretase inhibitors. Leukemia.

[71] Lewis H.D., Leveridge M., Strack P.R., Haldon C.D., O’Neil J., Kim H., Madin A., Hannam J.C., Look A.T., Kohl N. (2007). Apoptosis in T Cell Acute Lymphoblastic Leukemia Cells after Cell Cycle Arrest Induced by Pharmacological Inhibition of Notch Signaling. Chem. Biol..

[72] Tammam J., Ware C., Efferson C., O’Neil J., Rao S., Qu X., Gorenstein J., Angagaw M., Kim H., Kenific C. (2009). Down-regulation of the Notch pathway mediated by a γ-secretase inhibitor induces anti-tumour effects in mouse models of T-cell leukaemia. Br. J. Pharmacol..

[73] Vandersmissen C., Prieto C., Gielen O., Jacobs K., Nittner D., Maertens J., Segers H., Cools J. (2023). Combination therapy of a PSEN1-selective γ-secretase inhibitor with dexamethasone and an XPO1 inhibitor to target T-cell acute lymphoblastic leukemia. Haematologica.

[74] Real P.J., Tosello V., Palomero T., Castillo M., Hernando E., de Stanchina E., Sulis M.L., Barnes K., Sawai C., Homminga I. (2009). γ-secretase inhibitors reverse glucocorticoid resistance in T cell acute lymphoblastic leukemia. Nat. Med..

[75] Cullion K., Draheim K.M., Hermance N., Tammam J., Sharma V.M., Ware C., Nikov G., Krishnamoorthy V., Majumder P.K., Kelliher M.A. (2009). Targeting the Notch1 and mTOR pathways in a mouse T-ALL model. Blood.

[76] Borthakur G., Martinelli G., Raffoux E., Chevallier P., Chromik J., Lithio A., Smith C.L., Yuen E., Oakley G.J., Benhadji K.A. (2021). Phase 1 study to evaluate Crenigacestat (LY3039478) in combination with dexamethasone in patients with T-cell acute lymphoblastic leukemia and lymphoma. Cancer.

[77] Zweidler-McKay P.A., DeAngelo D.J., Douer D., Dombret H., Ottmann O.G., Vey N., Thomas D.A., Zhu L., Huang F., Bajaj G. (2014). The Safety and Activity of BMS-906024, a Gamma Secretase Inhibitor (GSI) with Anti-Notch Activity, in Patients with Relapsed T-Cell Acute Lymphoblastic Leukemia (T-ALL): Initial Results of a Phase 1 Trial. Blood.

[78] Habets R.A., de Bock C.E., Serneels L., Lodewijckx I., Verbeke D., Nittner D., Narlawar R., Demeyer S., Dooley J., Liston A. (2019). Safe targeting of T cell acute lymphoblastic leukemia by pathology-specific NOTCH inhibition. Sci. Transl. Med..

[79] Agnusdei V., Minuzzo S., Frasson C., Grassi A., Axelrod F., Satyal S., Gurney A., Hoey T., Seganfreddo E., Basso G. (2014). Therapeutic antibody targeting of Notch1 in T-acute lymphoblastic leukemia xenografts. Leukemia.

[80] Casulo C., Ruan J., Dang N.H., Gore L., Diefenbach C., Beaven A.W., Castro J.E., Porcu P., Faoro L., Dupont J. (2016). Safety and Preliminary Efficacy Results of a Phase I First-in-Human Study of the Novel Notch-1 Targeting Antibody Brontictuzumab (OMP-52M51) Administered Intravenously to Patients with Hematologic Malignancies. Blood.

[81] Minuzzo S., Agnusdei V., Pusceddu I., Pinazza M., Moserle L., Masiero M., Rossi E., Crescenzi M., Hoey T., Ponzoni M. (2014). DLL4 regulates NOTCH signaling and growth of T acute lymphoblastic leukemia cells in NOD/SCID mice. Carcinogenesis.

[82] Chiorean E.G., LoRusso P., Strother R.M., Diamond J.R., Younger A., Messersmith W.A., Adriaens L., Liu L., Kao R.J., DiCioccio A.T. (2015). A Phase I First-in-Human Study of Enoticumab (REGN421), a Fully Human Delta-like Ligand 4 (Dll4) Monoclonal Antibody in Patients with Advanced Solid Tumors. Clin. Cancer Res..

[83] Smith D.C., Eisenberg P.D., Manikhas G., Chugh R., Gubens M.A., Stagg R.J., Kapoun A.M., Xu L., Dupont J., Sikic B. (2014). A Phase I Dose Escalation and Expansion Study of the Anticancer Stem Cell Agent Demcizumab (Anti-DLL4) in Patients with Previously Treated Solid Tumors. Clin. Cancer Res..

[84] Roti G., Carlton A., Ross K.N., Markstein M., Pajcini K., Su A.H., Perrimon N., Pear W.S., Kung A.L., Blacklow S.C. (2013). Complementary Genomic Screens Identify SERCA as a Therapeutic Target in NOTCH1 Mutated Cancer. Cancer Cell.

[85] Marchesini M., Gherli A., Montanaro A., Patrizi L., Sorrentino C., Pagliaro L., Rompietti C., Kitara S., Heit S., Olesen C.E. (2020). Blockade of Oncogenic NOTCH1 with the SERCA Inhibitor CAD204520 in T Cell Acute Lymphoblastic Leukemia. Cell Chem. Biol..

[86] Koyama D., Kikuchi J., Hiraoka N., Wada T., Kurosawa H., Chiba S., Furukawa Y. (2014). Proteasome inhibitors exert cytotoxicity and increase chemosensitivity via transcriptional repression of Notch1 in T-cell acute lymphoblastic leukemia. Leukemia.

[87] Huang C., Hu X., Wang L., Lü S., Cheng H., Song X., Wang J., Yang J. (2012). Bortezomib suppresses the growth of leukemia cells with Notch1 overexpression in vivo and in vitro. Cancer Chemother. Pharmacol..

[88] Horton T.M., Whitlock J.A., Lu X., O’Brien M.M., Borowitz M.J., Devidas M., Raetz E.A., Brown P.A., Carroll W.L., Hunger S.P. (2019). Bortezomib reinduction chemotherapy in high-risk ALL in first relapse: A report from the Children’s Oncology Group. Br. J. Haematol..

[89] Teachey D.T., Devidas M., Wood B.L., Chen Z., Hayashi R.J., Hermiston M.L., Annett R.D., Archer J.H., Asselin B.L., August K.J. (2022). Children’s Oncology Group Trial AALL1231: A Phase III Clinical Trial Testing Bortezomib in Newly Diagnosed T-Cell Acute Lymphoblastic Leukemia and Lymphoma. J. Clin. Oncol..

[90] Moellering R.E., Cornejo M., Davis T.N., Bianco C.D., Aster J.C., Blacklow S.C., Kung A.L., Gilliland D.G., Verdine G.L., Bradner J.E. (2009). Direct inhibition of the NOTCH transcription factor complex. Nature.

[91] Astudillo L., Da Silva T.G., Wang Z., Han X., Jin K., VanWye J., Zhu X., Weaver K., Oashi T., Lopes P.E.M. (2016). The Small Molecule IMR-1 Inhibits the Notch Transcriptional Activation Complex to Suppress Tumorigenesis. Cancer Res..

[92] Lehal R., Zaric J., Vigolo M., Urech C., Frismantas V., Zangger N., Cao L., Berger A., Chicote I., Loubéry S. (2020). Pharmacological disruption of the Notch transcription factor complex. Proc. Natl. Acad. Sci. USA.

[93] Medinger M., Junker T., Heim D., Tzankov A., Jermann P.M., Bobadilla M., Vigolo M., Lehal R., Vogl F.D., Bauer M. (2022). CB-103: A novel CSL-NICD inhibitor for the treatment of NOTCH-driven T-cell acute lymphoblastic leukemia: A case report of complete clinical response in a patient with relapsed and refractory T-ALL. EJHaem.

[94] Hurtado C., Safarova A., Smith M., Chung R., Bruyneel A.A.N., Gomez-Galeno J., Oswald F., Larson C.J., Cashman J.R., Ruiz-Lozano P. (2019). Disruption of NOTCH signaling by a small molecule inhibitor of the transcription factor RBPJ. Sci. Rep..

